# Parotitis, Ending in Suppuration of the Glands and Death of the Patient

**Published:** 1846-12

**Authors:** C. A. Cowgill

**Affiliations:** The Resident Physicians at the Philadelphia Hospital


*Parotitis, ending in suppuration of the glands and death of the patient.* By C. A. Cowgill, M. D., one of the Resident Physicians at the Philadelphia Hospital.

Jacob Griener, German, aged 46 years, of a lymphatic temperament, admitted into the wards on the 20th of October, with slight horrors consequent upon drinking.

A few days afterwards, he was attacked with mild remittent fever, from which he was convalescent, though still weak, when on *Tuesday*, the third of November, he perceived a swelling at the angle of either jaw, which by *Wednesday*, had increased greatly in size, extending half an inch below the angle of the jaws, about the same distance backwards beneath the ears, and an inch and a half upon the cheeks. Slight pains were felt in the parts, which were very painful upon pressure. Pulse 100, small and quick. Thirty leeches were applied over each gland, followed by hot fomentations, and a saline laxative given.

*Thursday*. The glands not reduced in size; tenderness, however, greatly mitigated. Deglutition difficult. Gave the following

| | | |
|---|---|---|
| ℞. | Ant. et Potass. Tart. | gr. iss. |
| | Potass. Citrat. | ʒiss. |
| | Aquæ | ℥vj. |

Take ℥ss. every two hours.

*Friday*. The parotid glands are much larger than they were yesterday; hard and tender to the touch; deglutition gives much pain; general condition same as heretofore; applied 50 leeches over each gland.

*Saturday.* At this time, the most observable symptom was an effusion into the cellular tissue of the face and neck, so great as to close both eyes, and render deglutition almost impossible; some difficulty of respiration. Gave a cathartic of scammony and colocynth.

*Sunday.* The cathartic has operated several times, affording some relief, so that the patient can speak quite freely, and swallow without much difficulty; he is very weak. Give beef tea freely, and stop antiphlogistic measures.

*Monday.* The effusion has returned, and with it a semi-comatose condition. Blisters were placed upon the posterior portion of the cheeks, and behind the ears, but without avail, the patient dying in the evening.

*Autopsy.*

Thirty six hours after death: thoracic and abdominal viscera healthy; parotid glands twice their normal size, and each separate lobule in a state of suppuration. A suppurated lymphatic gland was discovered near each parotid; with this exception, surrounding tissues healthy. The longitudinal sinus of the brain contained much black, uncoagulated blood. Two ounces of a bloody serum found within the arachnoid, and half an ounce of a similar fluid in the ventricles; pia mater much injected; substance of brain healthy.

*November 16th,* 1846.

